# High seroprevalence of SARS-CoV-2 in cats linked to human infection in a Latin American country with elevated COVID-19 transmission and mortality

**DOI:** 10.3389/fvets.2025.1503000

**Published:** 2025-06-06

**Authors:** Alexandra Ulloa, Maritza Cordero-Ortiz, Luis M. Jara, Francesca Schiaffino, Cusi Ferradas, Camila Sánchez-Carrión, Ana Martínez-Vela, Jesús Hernández, Luis G. Giménez-Lirola

**Affiliations:** ^1^Facultad de Medicina Veterinaria y Zootecnia, Universidad Peruana Cayetano Heredia, Lima, Peru; ^2^Laboratorio de Inmunología, Centro de Investigación en Alimentación y Desarrollo, A.C., Hermosillo, Mexico; ^3^Unidad de Investigación en Enfermedades Emergentes y Cambio Climático (Emerge), Facultad de Salud Pública y Administración, Universidad Peruana Cayetano Heredia, Lima, Peru; ^4^Clinica Veterinaria Gatuario, Lima, Peru; ^5^Clinica Veterinaria Los Dominicos, Lima, Peru; ^6^Department of Veterinary Diagnostic and Production Animal Medicine, College of Veterinary Medicine, Iowa State University, Ames, IA, United States

**Keywords:** SARS-CoV-2, cats, COVID-19, seroprevalence, antibodies, ELISA

## Abstract

**Introduction:**

SARS-CoV-2 has been shown to infect various animal species, including companion animals such as cats. Although direct transmission from cats to humans has not been confirmed, monitoring the prevalence of infection in these animals remains critical as susceptible hosts to SARS-CoV-2, particularly in regions with high COVID-19 case numbers. This study aimed to evaluate the seroprevalence of SARS-CoV-2 in cats during the first wave of the COVID-19 pandemic in Lima, Peru.

**Methods:**

Serum samples from 544 cats, collected between 2020 and 2021, were tested for antibodies using a double antigen sandwich ELISA targeting the receptor-binding protein domain (RBD) and the nucleocapsid (N) proteins. This ELISA has a sensitivity of 80% and a specificity of 100%.

**Results:**

A seroprevalence of 43.8% was observed, with higher rates in females (63.9%), kittens (51.3%), and the Domestic Shorthair breed (93.2%). Among owners diagnosed with COVID-19, 95.5% of their cats were seropositive, with no cross-reactivity observed for other common feline diseases.

**Conclusion:**

These findings suggest significant SARS-CoV-2 exposure and possible infection in cats during the early pandemic phase in Peru. The high seroprevalence observed highlights the need for ongoing surveillance of companion animals, especially in regions with high human infection rates.

## Introduction

At the end of 2019, several cases of pneumonia of unknown origin were reported in Wuhan, China, with many initially associated with a local animal market (1). In January 2020, the World Health Organization (WHO) identified the causative agent as a novel betacoronavirus, Severe Acute Respiratory Syndrome coronavirus 2 (SARS-CoV-2), responsible for COVID-19. Since then, the pandemic has resulted in over 775 million cases and approximately 7 million deaths worldwide (2). Peru, in particular, has experienced one of the highest COVID-19-related mortality and case fatality rates worldwide (3). Early studies suggested that animals might act as reservoirs for the virus (4). SARS-CoV-2 has since been detected in various species, including zoo animals such as tigers and lions in the United States (5), as well exotic or domestic pets such as ferrets, cats, and dogs in China (6). Notably, cats have shown susceptibility to SARS-CoV-2 and may have the ability to transmit the virus (7).

Animal cases of SARS-CoV-2 infection have been documented globally, including in France (8), Spain (9), Italy (10), Belgium (11), Brazil (12), Argentina (13), Mexico (14, 15), and Peru (16).

Seroepidemiological studies are essential for understanding disease spread by detecting antibodies, which can reveal undiagnosed infections within a population (17). In Peru, a single serological study on a small population of domestic cats, whose owners reported COVID-19 symptoms, found a seropositivity rate of 31.7% (18). However, these findings require further investigation. Therefore, this study aimed to assess the seroprevalence of SARS-CoV-2 in domestic cats from Lima, Peru.

## Materials and methods

### Animals and samples

Serum samples were collected by convenience sampling from domestic cats at veterinary clinics in Lima, Peru, during the COVID-19 pandemic (2020–2021). Cats of various breeds, ages, origins, and sexes were included in the study if they required routine veterinary procedures, such as complete blood counts, serum biochemistry, or pre-surgical analysis. Data collected from each cat included age, sex, breed, location, and medical history (when veterinary care was sought due to illness). Owners were asked about COVID-19 exposure within the 2 weeks preceding the veterinary examination if the pets exhibited clinical signs, as determined by the veterinarians’ diagnostic criteria. Informed consent was obtained from the owners, who were provided with a detailed explanation of the study protocol. Serum or plasma was obtained from blood samples collected from cats, which were centrifuged at 447 × g for 5 min at room temperature (20–22°C). Aliquots of the serum were stored at −20°C until further analysis. Serum samples were excluded from the analysis if they exhibited excessive lipemia or hemolysis or lacked proper identification.

### Serology by double-antigen “sandwich” ELISA

A double-antigen “sandwich” ELISA was used to detect SARS-CoV-2 antibodies in mammals. This ELISA has a diagnostic sensitivity of 80% and a specificity of 100% (19). Microplates were coated with recombinant SARS-CoV-2 proteins nucleocapsid (N) and receptor-binding domain (RBD). These recombinant proteins were previously produced and evaluated in our laboratory (19). The genes used to express the N and RBD proteins were derived from the SARS-CoV-2 ancestral strain, ensuring their relevance to the early stages of the pandemic.

Samples and controls (positive and negative serum) were diluted 1:1, and 50 μL of each was added to corresponding wells for incubation at 37°C for 60 min with gentle shaking (30 rpm). After incubation, the solution was discarded, and the wells were washed five times with 250 μL of washing solution (0.1% PBS-Tween 20). The microtiter plate was then inverted on absorbent paper and gently tapped to ensure complete drying. Next, a solution containing RBD-horseradish peroxidase (HRP) and N-HRP antigens mixed in phosphate-buffered saline (PBS) was added to the wells and incubated for 30 min at RT with gentle agitation. After incubation, the solution was again discarded, and the wells were washed five times as previously described. To develop the color reaction, 3,3′,5,5′-tetramethylbenzidine (TMB) substrate was added to each well and incubated for 20 min at RT. The enzymatic reaction was halted by adding 50 μL of 1 M sulfuric acid to each well. The absorbance of the samples and controls was measured at 450 nm using an ELISA microplate reader (ELx800, Biotek, EEUU). Results were expressed as optical density (OD). Samples with OD values > 0.3615 were considered positive for SARS-CoV-2 antibodies, whereas samples with OD values < 0.32535 were considered negatives based on the Received Operating Characteristic (ROC) curves (19). The test was deemed valid if the average OD of positive controls was ≥ 0.3615 while negative controls remained below this threshold. Samples with OD values within 10% of the cut-off point (0.32535–0.3615) were classified as suspect or in the “gray zone” (20).

In addition, serum samples from three cats infected with feline coronavirus (infectious peritonitis), leukemia virus, and immunodeficiency virus were included for cross-reactivity testing. These samples were obtained from a pre-pandemic COVID-19 serum bank collected through routine veterinary clinical care, where a commercial ELISA was used for diagnosing common feline viral diseases.

### Statistical analysis

Frequency tables were generated to summarize the proportions of positive and negative samples based on age (<1 year: kitten, 1–10 years: adult, >10 years: senior), sex, breed, location, opitcal density and clinical signs using descriptive statistics (mean or median and standard deviation or interquartile range for continuous variables, and proportion and confidence intervals for categorical variables). To compare the differences among positive, suspect, and negative optical densities results, a Kruskal-Wallis test was employed. Chi-square and Fisher’s Exact Test were used to evaluate the association between sex and age with respect to positive and negative results. All statistical analyses were conducted using GraphPad Prism v. 9.5.

### Ethical considerations

The study was reviewed and approved by the Institutional Ethics Committee for the Use of Animals at the Universidad Peruana Cayetano Heredia, Peru (certificate number 207370). Informed consent was obtained from the owners for the collection of blood samples from their pets. Based on their diagnostic criteria, veterinarians asked owners about potential COVID-19 exposure in cats showing respiratory signs during the examination.

## Results

**Figure 1:**
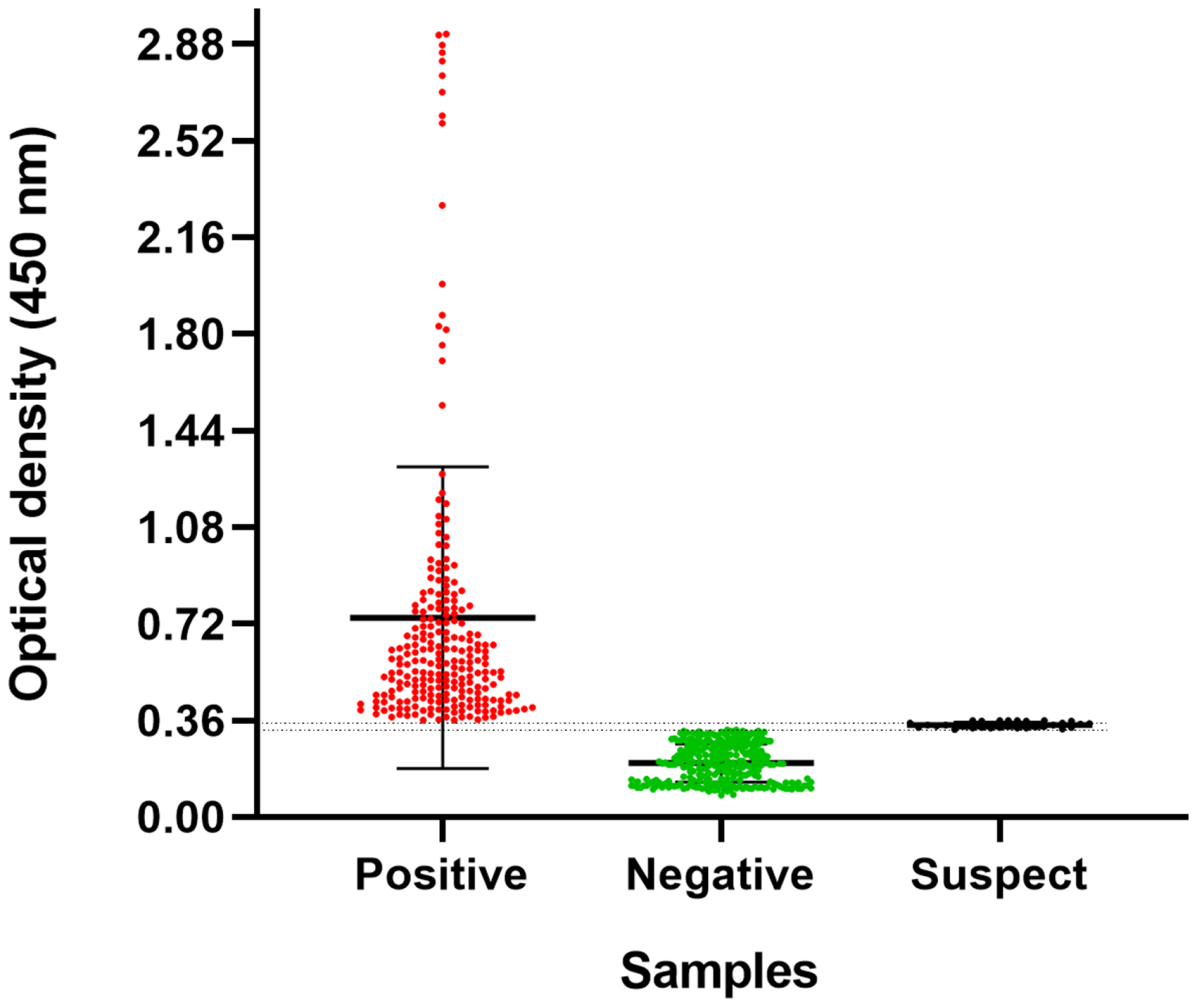
Optical densities of domestic cats (*n* = 544) using a double-antigen ELISA for SARS-CoV-2. Each point represents a serum sample from a cat. The cutoff point was determined based on ROC curves from previous standardization (19). The gray zone is indicated by two dotted lines (0.32535–0.3615), representing 10% below the cut-off OD (20). Error bars represent the median and interquartile range. A statistically significant difference was observed between the optical densities of the groups (*p* < 0.0001), as determined by the Kruskal-Wallis test.

**Figure 2:**
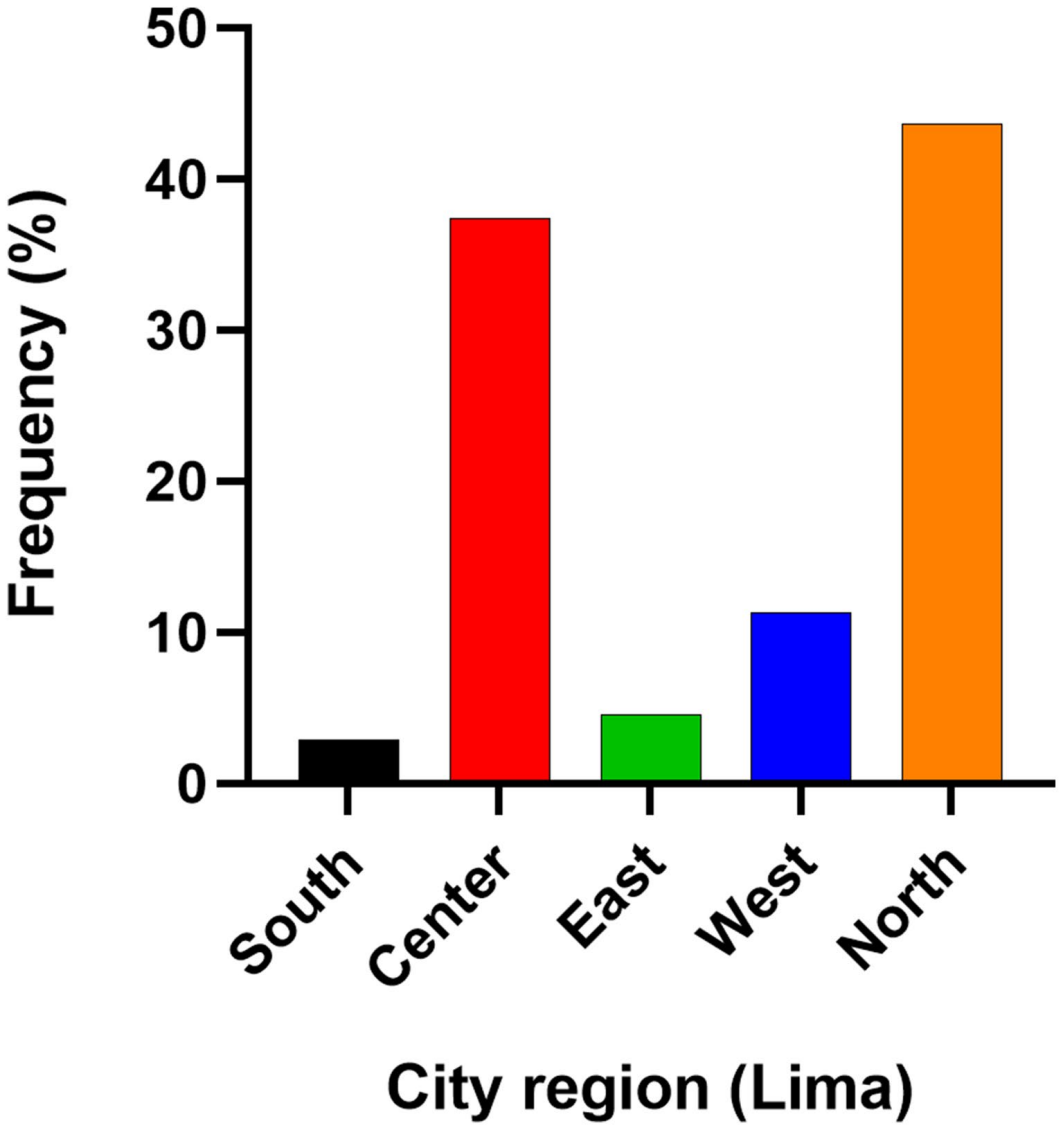
Distribution of positive cat samples (*n* = 238) by sampling location in Lima, Peru. Lima, the capital of Peru, is divided into 49 districts, organized geographycally into regions such as north, center, east, and west. Frequency was calculated as the number of positive cases in each region divided by the total of positive cases.

Of the 700 collected samples, 544 (211 males, 333 females, 279 kittens, 217 adults, and 48 seniors) met the inclusion criteria and were processed. Among these, 43.8% (238/544) (CI: 95%: 39.5–48) were positive for SARS-CoV-2 antibodies, 50.7% (276/544) (CI: 95%: 46.5–55) were negative, and 5.5% (30/544) (CI: 95%: 3.7–7.8) were classified as suspect. The median OD value in the positive group was 0.72, with some samples exhibiting high reactivity, showing values up to four times higher than the cut-off. The distribution of optical density values is shown in Figure 1. The three serum samples positive for feline leukemia virus, feline coronavirus, and feline immunodeficiency virus were all negative in the cross-reaction test for SARS-CoV-2. Geographic analysis revealed that positive cases were most prevalent in the northern region of Lima, accounting for 43.7% (104/238) of cases, followed by the central region at 37.4% (89/238) (Figure 2).

Analysis revealed that 51.3% (122/238) of the positive cats were kittens, while 40.3% (96/238) were adults; however, there was no significant association between age and SARS-CoV-2 seropositivity (*p* > 0.05). Additionally, 63.9% (152/238) of the positive samples were females, with no significant correlation to positivity (*p* > 0.05). The Domestic Shorthair (DHS) breed accounted for 93.3% (222/238) of the positive samples.

Twenty-two owners with a history of COVID-19 were recorded. Their cats exhibited a seropositivity of 95.5% (21/22). Of 238 seropositive cats, only 15.6% (37/238) showed clinical signs. Notably, 32.4% (12/37) of these cats had owners with a COVID-19 history. In the remaining seropositive cats with clinical signs (25/37, 70.3%), no recorded history of COVID-19 in their owners was available.

The median age of seropositive cats with clinical signs was 3 years, with 54.05% (20/37) being males. Domestic Shorthair (DSH) cats had the highest seroprevalence, accounting for 72.97% (27/37) of cases. Clinical signs observed in seropositive cats included respiratory signs, with sneezing, coughing, and nasal discharge being the most common. Among these cases, three animals were in critical condition, exhibiting multiple clinical signs (Table 1). The clinical signs observed in seropositive cats included respiratory signs, with the most common being sneezing, coughing, and nasal secretions. Among these cases, three animals were in critical condition, exhibiting multiple clinical signs.

**Table 1:** Characteristics of the symptomatic cats analyzed for SARS-CoV-2 seroprevalence in Lima, Peru.

Age (years)	Sex	Breed	Symptoms (cats) and history of COVID-19 (owner)	ELISA OD
0.9	M	DSH	Enteritis	1.986
7	F	DSH	Respiratory distress. Owner with COVID-19	2.613
16	M	DSH	Respiratory distress	2.848
13	M	DSH	Weakness, anorexia	1.183
0.8	F	DSH	Lymphadenopathy	0.629
0.5	M	Bengal	Watery eyes, sneezing. Owner with COVID-19	0.6
3	F	DSH	Weakness	0.469
10	M	DSH	Respiratory distress, nasal discharge, sneezing, chylothorax. Owner with COVID-19	0.531
4	M	DSH	Urinary tract infection	0.928
4	M	DSH	Urethral obstruction	0.619
9	M	DSH	Weakness, sneezing, nasal discharge	0.595
3	F	DSH	Hyporrexia	0.435
0.5	F	Russian Blue	Weakness, nasal discharge. Owner with COVID-19	0.573
9	M	DSH	Weakness, inappetence, pulmonary edema	0.689
4	F	Maine Coon	Bronchitis, asthma, sudden death. Owner with COVID-19	0.684
12	F	Siamese	Dyspnea, pulmonary edema	0.399
1	M	DSH	Weakness, anorexia	1.167
8	F	DSH	Weakness	0.73
3	F	DSH	Inappetence, fever	0.436
4	M	DSH	Weakness	0.493
3	F	DSH	Weakness, immundeficiency	1.758
4	M	DSH	Weakness	0.39
1	M	Bengal	Sneezing, cough. Owner with COVID-19	1.87
0.7	F	DSH	Sneezing, eye discharge	0.624
0.7	M	DSH	Cough, fever, weakness. Owner with COVID-19	0.417
0.8	M	DSH	Bronchitis	0.539
1	F	DSH	Weakness	0.61
4	M	Persian	Weakness, inappetence, vomiting	0.809
0.11	F	Persian	Anorexia	0.676
4	M	Russian Blue	Respiratory distress	0.739
6	F	Russian Blue	Sneezing. Owner with COVID-19	1.11
6	M	DSH	Asthma	0.642
1	M	Bengal	Weakness. Owner with COVID-19	0.722
0.5	M	DSH	Inappetence, fever. Owner with COVID-19	0.661
0.5	F	DSH	Sneezing. Owner with COVID-19	0.373
0.4	F	DSH	Immunodeficiency	0.432
2	F	DSH	Respiratory distress. Owner with COVID-19	0.678

M, male; F, female; OD, optical density; DSH, Domestic Shorthair cat.

## Discussion

Since the onset of the COVID-19 pandemic, cats have been identified as susceptible to SARS-CoV-2 infection, likely due to the similarity of their ACE2 receptor to that of humans and their close contact with infected owners (21). Although transmission from domestic cats to humans has not been documented, ongoing monitoring of infections in various animal species is essential to understand SARS-CoV-2 potential for adaption and circulation in new hosts, especially with the emergence of new variants (22).

The initial reports of pet infections, including those in cats, occurred during the first wave of COVID-19 (10, 23, 24), and Peru recorded one of the highest human case counts globally (3). Despite this, studies assessing SARS-CoV-2 infection in cats, particularly in areas with high COVID-19 rates using serological assays, remain limited. Understanding transmission dynamics and susceptibility in cats is critical, giving their high seroprevalence in many regions (11, 25). Therefore, this study aimed to evaluate the SARS-CoV-2 seroprevalence in cats from 2020 to 2021 during the first and second pandemic waves in Peru.

A double-antigen sandwich ELISA utilizing SARS-CoV-2 RBD and N proteins was used. This assay has previously demonstrated robust performance in detecting all anti-SARS-CoV-2 antibody isotypes across several species, including cats, rats, dogs, and tiggers, and has shown strong concordance with microneutralization assay (19). This broader detection of both neutralizing and non-neutralizing antibodies offers a reflection of overall exposure to SARS-CoV-2. Moreover, the seropositive results align with the clinical signs observed in many cats from COVID-19 patients in our study, corroborating similar findings from other studies (21, 26, 27). Given that microneutralization assays may not alwasys be readily available, especially in regions like Peru, the double-antigen sandwich ELISA offers a reliable alternative for seroprevalence studies.

The present study reveal a high seropositivity rate; however, a limitation was that clinical data were only available for a subset of cats. The clinical signs observed in seropositive animals were consistent with those associated with SARS-CoV-2 infection in cats, such as coughing, sneezing, and dyspnea (28). In natural infections, up to 42% of cats that develop clinical signs may test seropositive, although seropositivity is not associated with the presence of one or more clinical signs (29). In contrast, experimental infections are typically asymptomatic, although transmission between cats is still possible (30). The high OD values in ELISA of seropositive cats displaying clinical signs suggest an immune response indicative of active or recent SARS-CoV-2 infection. A significant proportion of cats belonging to confirmed COVID-19-positive owners further support the association between human and feline infections.

Interestingly, some positive samples (symptomatic cats) with a low OD value and another with a high OD were linked to households with COVID-19, suggesting that the low positives may reflect true SARS-CoV-2 infections in cats rather than cross-reactivity with other coronaviruses (21, 26, 27). While cross-reactivity against feline coronaviruses has been observed in some studies, the absence of significant serologic cross-reactivity against SARS-CoV-2 has been reported (31), and low specificity in ELISAs related to the N protein, but not to the RBD, has been observed (32, 33).

Although the methodology in this study was similar to previous reports, a higher positivity rate was observed in females compared to males, consistent with earlier findings (34, 35). However, other research reported no significant sex differences (9). Experimental studies have indicated increased susceptibility to infection in younger cats (6), a finding supported by other serological studies (34). Other studies have not reported a significantly higher risk in older pets, contrasting with our results (14). Furthermore, positive samples were predominantly originated from Domestic Shorthair breed, which aligns with trends observed in other studies (14, 35).

Previous reports has confirmed the presence of neutralizing antibodies against SARS-CoV-2 in some cats in Peru (18). This study enhanced the sample size and identified a notably high seroprevalence, similar to that reported in other countries like the United States (25), while other studies have indicated seropositivity rates around 20% (11, 36). The elevated seroprevalence observed in this study, coupled with the high COVID-19 incidence in the Peruvian population, may be attributed to factors such as population density (including human and animal ratios), and the lack of effectiveness of control measures during the pandemic. Interestingly, the highest seropositivities in our study were found in the northern and central zones of Lima. This coincides with the districts in these regions that had the highest frequency and attack rates of COVID-19 in humans during 2020–2021, suggesting that cats in these areas may have been at higher risk of exposure to the virus.

This study has several limitations. First, the convenience sampling method may not fully represent the broader cat population in Lima, limiting the generalizability of the results. Additionally, the lack of follow-up testing, such as PCR or neutralization assays, prevented confirmation of active infection and a more detailed assessment of antibody responses over time. This limitation also restricts our ability to draw direct correlations between seroprevalence and clinical disease. Furthermore, limited information on the clinical signs of the cats, especially asymptomatic cases, hinders a comprehensive understanding of disease manifestation in relation to seropositivity. The absence of detailed data regarding the owners’ COVID-19 status also prevents a more precise determination of potential exposure risk factors for the cats. Despite these limitations, this study provides valuable data on SARS-CoV-2 exposure in domestic cats, particularly in regions with high human infection rates.

These findings are valuable for informing new epidemiological surveillance programs that utilize serological tools under the One Health framework. This approach is crucial for understanding the transmission dynamics of SARS-CoV-2 and other emerging viruses, not only in humans but also in companion animals such as cats, ferrets, and other susceptible species. Integrating pets into this framework will enhance our understanding of how zoonotic pathogens spread and inform more effective monitoring strategies.

## Conclusion

This study, to our knowledge, reports the highest seroprevalence of SARS-CoV-2 in cats globaly during the 2020–2021 pandemic. Although susceptibility based on sex and age was not found to be significant and remains a subject of ongoing debate, the high seroprevalence observed highlights the importance of continuous surveillance of companion animals, particularly in regions with high human infection rates. The strong relation between feline seropositivity and owner infection further emphasizes the relevance of a One Health approach in understanding the dynamics of virus transmission and in guiding future epidemiological monitoring efforts.

## Data Availability

The raw data supporting the conclusions of this article will be made available by the authors, without undue reservation.
